# Dysregulation of Neutrophil–Endothelial Communication in Sepsis: Mechanisms and Therapeutic Perspectives

**DOI:** 10.3390/cells15070581

**Published:** 2026-03-25

**Authors:** Nazgol Esmalian Afyouni, Mohammad F. Kiani, Laurie E. Kilpatrick

**Affiliations:** 1 Center for Inflammation and Lung Research, Department of Microbiology, Immunology and Inflammation, Lewis Katz School of Medicine, Temple University, Philadelphia, PA 19140, USA; nazgol.afyouni@temple.edu; 2 Department of Mechanical Engineering, Temple University, Philadelphia, PA 19122, USA; mohammad.kiani@temple.edu; 3 Department of Radiation Oncology, Lewis Katz School of Medicine, Temple University, Philadelphia, PA 19140, USA

**Keywords:** sepsis, neutrophil–endothelial interaction, neutrophil migration, reverse-migrated neutrophils, omics, microphysiological assays

## Abstract

Sepsis is a clinical syndrome defined as life-threatening organ dysfunction caused by a dysregulation in immune response to infection. Dysregulated neutrophil activity plays a critical role in sepsis-induced organ failure through interactions with the vascular endothelial cells during forward and reverse migration, resulting in vascular barrier disruption and increased neutrophil trafficking into vital organs. Therapeutic approaches for treating sepsis are mainly supportive. Due to limited clinical translation from rodent models, complexity of the pathophysiology, and most importantly, the heterogenous nature of sepsis, no significant therapeutics have been successfully developed to address the underlying immune dysregulation. In this review, we will discuss the important gap in knowledge on the fundamental mechanisms of neutrophil–endothelial interaction, the role that neutrophil forward and reverse migration plays in organ damage in sepsis, and how neutrophil and endothelial cell heterogeneity impact cell–cell communication. We will explore emerging methodologies, including novel omic and microphysiological systems, to study the underlying mechanism of neutrophil–endothelial interaction and neutrophil forward migration/reverse migration. Finally, we will review potential therapeutic targets modulating neutrophil–endothelial interaction and the challenges of translating them from bench to bedside.

## 1. Introduction

Sepsis is defined as “life-threatening organ dysfunction caused by a dysregulated host response to infection” according to The Third International Consensus Definitions for Sepsis and Septic Shock (Sepsis-3) [1]. Sepsis remains a significant global health challenge contributing to high rates of morbidity, mortality, and healthcare expenses. The global incidence of sepsis is significant, with an annual incidence of 47–50 million cases/year resulting in more than 11 million deaths [2]. In the US alone, there are over 1.7 million cases/year and greater than 350,000 deaths. Between 2019 and 2021, sepsis mortality increased significantly as a result of COVID-19 infections [3]. Sepsis is one of the leading causes of death in hospitals, with healthcare costs amounting to over $60 billion dollars/year. Demographic studies show that young children, the elderly, and those with chronic medical conditions are at higher risk for sepsis and increased mortality [2].

In sepsis, neutrophils are important contributors to dysregulated immune response and play a critical role in sepsis-induced organ failure through interactions with the vascular endothelium resulting in barrier disruption and increased neutrophil trafficking into vital organs, leading to tissue damage and organ failure [4,5,6]. Therapeutic approaches are largely supportive, and no drugs are available that target the complicated immune dysregulation and the heterogenous pathogenesis of sepsis [7,8,9,10,11,12]. There is now a consensus that the host response to sepsis is highly diverse among patients, and this heterogeneity impacts immune function and response to infection [9,10,12,13,14,15,16,17,18,19,20]. Thus, these diverse responses may explain the inconsistency in response to immunomodulating treatments and failures of clinical trials.

While neutrophils are critical to host defense, neutrophil dysregulation and inappropriate release of inflammatory mediators, proteases, reactive oxygen species (ROS), and neutrophil extracellular traps (NETs) can damage endothelial cells, leading to organ injury [4,21,22]. Conversely, neutrophil immunosuppression is also reported in sepsis patients [22,23,24,25], while some patients develop a mixed status with characteristics of persistent inflammation and immunosuppression [26,27]. Multiple neutrophil subpopulations that differ in cell markers, protein expression, and function are present in sepsis patients exhibiting proinflammatory or immune suppressive characteristics [28,29]. Conventional or high-density neutrophils (HDNs) are well characterized, but additional subpopulations of low-density neutrophils (LDNs) are also observed in sepsis patients with distinct biological characteristics compared to conventional HDNs [28,29,30,31]. Furthermore, recent studies indicate that some neutrophil subpopulations which have migrated into tissues can undergo a process of reverse migration and can traverse the vascular endothelium and return to the circulation. Both proinflammatory and protective mechanisms have been reported, and the role of reverse-migrated neutrophils appears to be context-dependent [32,33,34,35,36,37].

With the identification of multiple neutrophil phenotypes in sepsis, it is important to characterize fundamental mechanisms and ascertain the impact of these diverse neutrophil phenotypes on interactions with the vascular endothelium, barrier permeability, and neutrophil trafficking, including forward and reverse migration. Mechanistic insight into these different neutrophil–endothelial cell interactions may lead to the identification of new therapeutic targets [38,39,40]. In this review, we focus on the interaction of neutrophils and endothelial cells, highlighting the critical changes occurring in sepsis, including endothelial cells activation, neutrophil adhesion/migration, mechanisms of reverse-migrated neutrophils, alterations in barrier permeability, and endothelial cell damage. We will also discuss emerging methodologies to investigate neutrophil–endothelial cell interactions and to identify potential novel therapeutics targeting these interactions in sepsis.

## 2. Endothelial Cell Function and Heterogeneity

The vascular endothelium is composed of a single layer of cells which act as the primary interface between the bloodstream and surrounding tissues. These cells communicate with other cells through multiple physiological functions, including immunologic responses and cell surface protein expression, as well as secretory and metabolic activities [41,42,43,44]. Endothelial cells are covered with a layer of glycosaminoglycan, glycoproteins, and proteoglycans, known as the glycocalyx, which plays a critical role in endothelial cell integrity and interaction with immune cells [44,45]. Endothelial cells react to both biophysical (such as shear stress) and biochemical (such as cytokines and ROS) stimuli [45,46].

**Figure 1:**
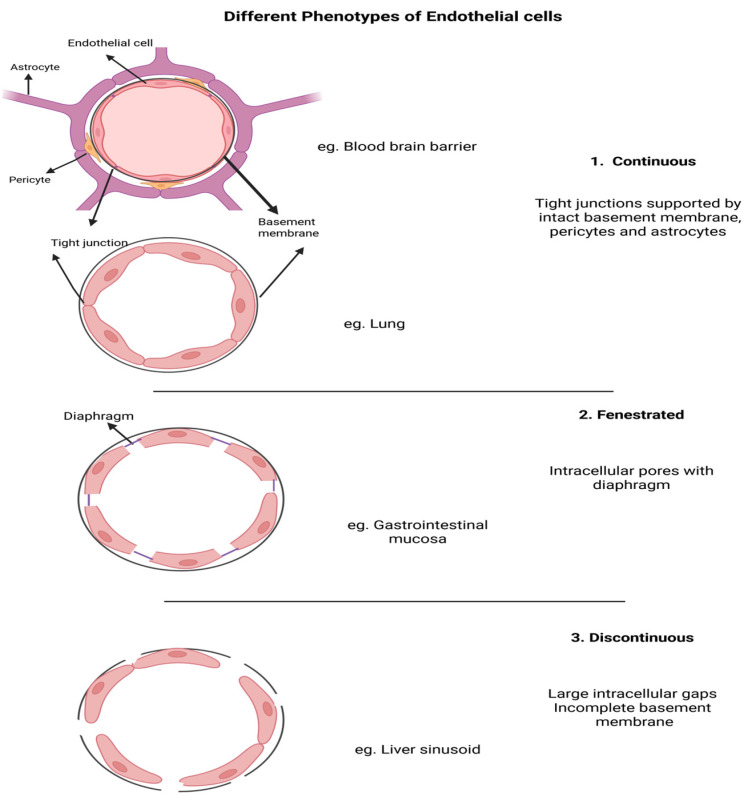
Different phenotypes of endothelial cells. Capillary endothelial cells are classified as 1. continuous, 2. fenestrated, and 3. discontinuous. As an example of the continuous phenotype, endothelial cells of the blood–brain barrier (BBB) have increased tight junctions linking endothelial cells firmly together, supported by pericytes and astrocytes. Fenestrated endothelial cells have intracellular pores with a diaphragm in endocrine and intestinal mucosa, but the diaphragm is absent in kidney glomeruli endothelial cells. Discontinuous endothelial cells can be found in the liver and spleen and have large pores between the cells, lacking a complete basement membrane. Created in https://BioRender.com.

Organ-specific microvascular endothelial cell heterogeneity is well established, with different phenotypes specialized to support cellular and metabolic demands of specific organs by regulating various aspects of solute and nutrient transport. Vascular endothelial cells are structurally classified as 1. continuous, 2. fenestrated, and 3. discontinuous (Figure 1) [45,47]. Endothelial cells displaying the continuous phenotype are characterized by an increased number of tight junctions between cells, resulting in low permeability to solutes and proteins. Continuous endothelial cells are primarily located in the microvasculature of the heart, brain, and lung [48,49]. In contrast, fenestrated endothelial cells are distinguished by small pores or fenestrations with a diaphragm that penetrates the endothelium. Fenestrated endothelial cells are located in the choroid plexus, gastrointestinal mucosa, and kidney glomeruli, but kidney endothelial cells lack a diaphragm. Compared to the continuous phenotype, fenestrations allow for increased permeability, where rapid exchange of substances is important for absorption, filtration, and secretion [50]. Endothelial cells with the discontinuous phenotype are found in the liver and spleen and lack a continuous endothelial layer and diaphragm and contain an incomplete basement membrane, resulting in larger gaps between cells than in fenestrated cells. This structure permits enhanced permeability and exchange of cells and larger proteins and has an important role in immune responses and filtration [48,51,52]. These organ-specific variations in endothelial cell structure and function result in different mechanisms of neutrophil–endothelial cell interactions.

## 3. Neutrophil Function and Heterogeneity

Neutrophils are key cells in the innate immune system and are critical to host defense. Acting through multiple mechanisms in response to pathogens or inflammation, neutrophils can phagocytose microorganisms and kill pathogens through the release of reactive oxygen and nitrogen species, as well as granule components, including elastase, myeloperoxidase, lactoferrin and matrix metalloproteinase, cathepsins, defensins, and lysozyme [53,54]. Additionally, during activation, neutrophils produce neutrophil extracellular traps (NETs) by extruding web-like structures composed of chromatin DNA, histones, and granular proteins that can neutralize microorganisms and inhibit their spread [55,56]. Mature neutrophils are identified by key cell surface markers that include high expression of cluster of differentiation (CD) markers CD10, CD16, CD62L, and the chemokine receptor CXCR2. Mature neutrophils are also characterized by low expression of the chemokine receptor CXCR4. These cells contain multi-lobular nuclei and multiple types of granules, including azurophilic, specific, tertiary, and secretory, with specific function in antimicrobial and immune functions [57,58,59].

During sepsis, neutrophils are the first responders to sites of infection and inflammation and are the first immune cells to migrate into organs. In response to inflammatory signals, increased numbers of neutrophils are released from the bone marrow, increasing the numbers of circulating neutrophils. As the disease progresses through a process of emergency myelopoiesis, immature neutrophils are released from the bone marrow and characterized by CD15 and CD11b expression and low expression of CD10, CD16, and CD62L but high expression of CXCR4 [60]. Immature neutrophils are functionally different from mature neutrophils, with impaired chemotaxis and killing and increased release of proinflammatory cytokines such as TNF.

The immune response becomes dysregulated in sepsis, and inappropriate release of inflammatory mediators, proteases, and ROS can damage endothelial cells, leading to organ injury [4,21,22]. Immunocompromised neutrophils with impaired function have also been reported in some sepsis patients [4,16,22,23,24]; other patients develop a mixed response with characteristics of continuing inflammation and immunosuppression [26,27]. Neutrophil populations are heterogeneous, and multiple neutrophil subpopulations that differ in function and protein expression are present in individuals; these subpopulations vary in healthy individuals and in sepsis patients [28,29].

Conventional or high-density neutrophils (HDNs) are well characterized, but an additional subpopulation of low-density neutrophils (LDNs) is detected in sepsis patients, with distinct functional characteristics compared to conventional HDNs [28,31]. LDNs, which co-localize with peripheral blood mononuclear cells in density gradient centrifugation, significantly contribute to the immune response in sepsis. LDNs are characterized as proinflammatory or immunosuppressive depending on the microenvironment and disease pathology [29,30,61]. In sepsis, LDNs comprise a significant percentage of the neutrophil population with elevated CXCR4 expression—which regulates bone marrow release—compared to HDNs, suggesting that LDNs are at a different life stage, highlighting neutrophil heterogeneity [28,62]. LDNs have high expression of HDN surface markers, as well as markers of activated neutrophils, including CD15, CD16, CD11b, and CD66b [61,63]. These cells can have unique characteristics, including decreased enzymatic activity, phagocytic function, chemotactic ability, and composition of their intracellular granules, suggesting reduced ability to clear pathogens, as well as delayed apoptosis and elevated formation of NETs, indicating impaired function [28,64]. For example, a study on patients with COVID-19-induced sepsis suggested an important contribution of an LDN subset that expressed intermediate levels of CD16 and demonstrated proinflammatory expression with enhanced cytokine production and NET formation. The authors concluded that this LDN subset contributed to the development of systemic inflammatory response syndrome (SIRS) and acute respiratory distress syndrome (ARDS) in these patients [65]. However, in healthy individuals, LDNs demonstrate no difference in ROS production and rate of apoptosis as compared to HDNs [66]. While decreased chemotaxis activity of LDNs implies an anti-inflammatory characteristic, other characteristics, such as delayed apoptosis in the setting of inflammatory disease, suggest a potentially proinflammatory response. In sepsis, detailed functional characteristics of these different neutrophil subpopulations and their interactions with the endothelium have not been delineated.

Neutrophil heterogenicity extends beyond differences in cell density, as these cells display diverse phenotypes and functional profiles depending on their maturation state and activation status in both health and disease conditions [67]. Several studies suggest variable neutrophil phenotypes in sepsis [57,68]. Numerous techniques have been employed to characterize different neutrophil cell types through morphology-based assays, flow cytometry, mass cytometry, bulk transcriptomics, single-cell transcriptomics, and cell functional studies [58,59]. Using these different technologies, several unique neutrophil subpopulations have been reported in sepsis patients, in addition to LDN and immature neutrophils with different markers and functional parameters that can be proinflammatory or immunosuppressive. Some examples of identified proinflammatory subtypes include proinflammatory neutrophils (N1) that release high levels of proinflammatory cytokines; aged neutrophils which have an extended lifespan, increased CXCR4 expression, enhanced phagocytosis, and increased migration; and neutrophils that express ICAM1+s which demonstrate enhanced phagocytosis and production of ROS and NETs [57]. Multiple immunosuppressive neutrophils have been characterized, including N2 neutrophils that are categorized by expression of PD-L1 (programmed death ligand-1) and arginase and that can suppress T cell function, leading to immunosuppression. Other immunosuppressive phenotypes include neutrophils with augmented expression of OLFM4+. OLFM4+ neutrophils have decreased bactericidal activity and are elevated in sepsis patients with a higher risk for organ failure and death [19]. With the availability of omics analysis and single-cell RNA sequencing, identification of distinct neutrophil subpopulations has been greatly enhanced, increasing our understanding of neutrophil heterogeneity. For example, a study using single-cell and gene expression data of sepsis patients categorized neutrophils into five distinct subsets based on expressed cellular markers: colony stimulating factor 3 receptor (CSF3R) neutrophils, matrix metalloproteinase-9 (MMP9) neutrophils, lysozyme C (LYZ) neutrophils, S100A9 neutrophils, and cystatin 3 neutrophils. The proportion of CSF3R neutrophils and LYZ neutrophils was reduced in the sepsis group compared to the healthy control group [69,70]. In another study, a neutrophil phenotype characterized by poor migratory efficiency, decreased NET formation, and prolonged neutrophil survival was reported in sepsis patients with metabolic acidosis [71]. Our group identified unique neutrophil phenotypes in patients with severe sepsis based on their ex vivo functional properties [38]. These functional phenotypes had distinct proteomic signatures which differentiated sepsis patients by important clinical parameters related to disease severity. Thus, multiple neutrophil subpopulations that differ in cell markers, protein expression, and functional properties have been reported in sepsis patients using a variety of techniques from omics, plasma biomarkers, and variations in neutrophil function [28,31]. Whether neutrophil heterogeneity in sepsis is dependent on disease stage and evolves over time or is an intrinsic property of patients’ immune function and reflects the heterogeneity and diversity of host responses is not known. Furthermore, overlap in these different reported phenotypes has not been examined to date. How these diverse phenotypes impact neutrophil–endothelial cell interaction and neutrophil trafficking has also not been studied in detail. Nonetheless, these diverse neutrophil phenotypes may explain the inconsistency in response to immunomodulating treatments and the failures of clinical trials in sepsis patients.

## 4. Neutrophil–Endothelial Cell Interactions and the Neutrophil Extravasation Cascade in Sepsis: Impact of Heterogeneity

Neutrophil–endothelial cell interactions in sepsis have a critical role in the pathogenesis of organ failure due to disruption of the vascular barrier, micro-thrombosis formation, and exacerbation of the inflammatory response [64,72,73]. These specific interactions are influenced by organ-specific endothelial cell phenotypes, as well as neutrophil heterogeneity [38,73]. During sepsis, the endothelium is an active participant in the inflammatory response. Activation of endothelial cells enhances crosstalk between immune cells and endothelial cells and can induce endothelial cells to reprogram toward a proapoptotic, proinflammatory, prothrombotic, pro-adhesive phenotype [74,75]. Variations in neutrophil function resulting from neutrophil heterogeneity from sepsis patients, as compared to neutrophils from healthy individuals, also impact the interaction with endothelial cells [29,76,77,78,79,80,81].

**Figure 2:**
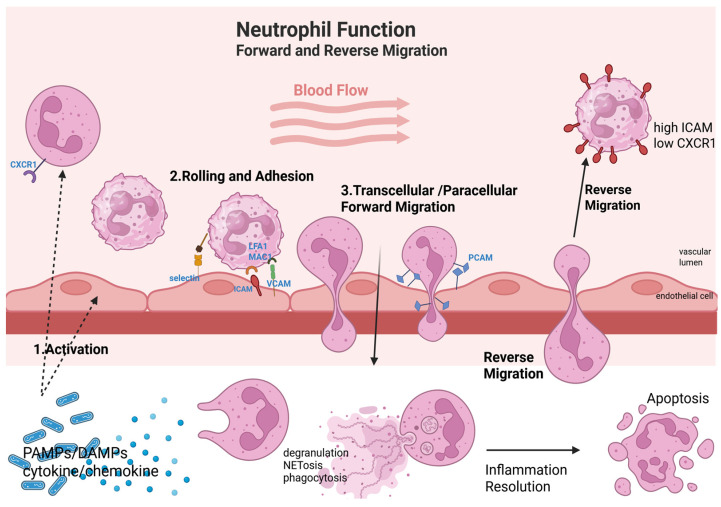
Neutrophil function, forward and reverse migration. 1. Activation of neutrophils and endothelial cells is stimulated by PAMPs (such as LPS, bacterial nucleic acids, and peptidoglycan) and DAMPs (intracellular components such as proteins and ribonucleic acid that are released through cellular damage). 2. Rolling and adhesion of neutrophils across endothelial cells barrier occur via interaction of their surface molecules. 3. Neutrophils extravasate via paracellular or transcellular pathways. Neutrophils are guided by a chemoattractant gradient in the tissue, where they act by degranulation, phagocytosis, and NET formation; finally, they undergo apoptosis or reverse migration back to the blood stream, with alterations in the cell surface molecules. Created in https://BioRender.com.

Neutrophils reach sites of inflammation by migrating through the endothelial barrier in a multi-step process known as the neutrophil extravasation cascade (Figure 2) [82]. Neutrophil extravasation is a highly orchestrated process involving sequential interactions between surface molecules and shear forces [83]. During sepsis, PAMPs (pathogen-associated molecular patterns)—such as LPS, bacterial nucleic acids, and peptidoglycan—and DAMPs (damage-associated molecular patterns) which are intracellular components, such as proteins and ribonucleic acid, that are released through cellular damage, as well as other proinflammatory mediators, activate both neutrophils and endothelial cells. PAMPs and DAMPs bind to pattern recognition receptors (PRRs) on both cell types and activate multiple signaling pathways, including toll-like receptors (TLRs) and activation of proinflammatory transcription factors such as NFκB, leading to the release of cytokines (TNF, IL-6, IL-1β), chemokines (such as IL-8, CCL2), and inflammatory mediators. In endothelial cells, PAMPs and DAMPs trigger the upregulation of adhesion molecules (E-selectin, ICAM-1, VCAM-1) that are important in neutrophil adhesion [82,84]. Neutrophil attachment to endothelial cells is mediated by transient interactions between endothelial selectins (E-selectin and P-selectin) and their glycoprotein ligands on neutrophils such as P-selectin glycoprotein ligand (PSGL-1) [85,86,87]. These weak bonds allow neutrophils to roll along the vascular endothelium under shear stress [88]. In the process of rolling, the chemokine CXCL12 guides neutrophils via interaction with the CXC chemokine receptor CXCR4 [89]. Selectin-mediated interactions also trigger other intracellular signaling pathways, involving tyrosine phosphorylation and mitogen activated protein kinase (MAPK) activation, which results in integrin activation and clustering of adhesion molecules on the cell surface [90]. Firm adhesion follows, mediated by integrins, including lymphocyte function-associated antigen-1 (LFA-1, CD11a/CD18), very late antigen-4 (VLA-4, CD49d/CD29), and Mac-1 (CD11b/CD18), on neutrophils, which bind to the adhesion molecules ICAM-1, ICAM-2, and VCAM-1 [91]. Two signaling pathways are involved in the conformational change of CD11a/CD18, enabling it to transiently bind to ICAM-1: one pathway is phosphatidylinositol 3 kinase (PI3K)-dependent and P-Rex-1-dependent and the other involves p38 MAPK and Ras-related protein [90]. This step is enhanced by chemokine signaling, as endothelial cells present chemokines like CXCL8 on their surface via glycosaminoglycans, stimulating CXCR1 and CXCR2 receptors on neutrophils, leading to integrin activation [88,92]. Subsequently, neutrophils undergo spreading and transmigration (Figure 2) [88,92]. During sepsis, the expression of β1 and/or β2 integrins on neutrophils and of ICAM-1 and VCAM-1 on endothelial cells is often upregulated, promoting strong adhesion between neutrophils and the vascular endothelium [93,94].

Neutrophils cross the endothelial barrier by paracellular (between endothelial cells) or transcellular (through endothelial cells) routes (Figure 2). Neutrophils preferentially migrate via paracellular transmigration, which occurs with engagement of endothelial ICAM1/2 VCAM-1, JAM-C (junctional adhesion molecules), PECAM-1(platelet endothelial cell adhesion molecule), CD99, and neutrophil-selective adhesion molecules. Moreover, during paracellular diapedesis, VE-cadherin (an endothelial-specific adhesion molecule located at endothelial cell junctions) is phosphorylated and physically displaced from adherens junctions. During transcellular migration, a transcellular channel between the apical and basal membrane of endothelial cells is formed, with significant roles of ICAM-1, PECAM-1, CD99, and JAM-A. In this pathway, neutrophils pass through endothelial cells, leaving the endothelial cell junctions intact [25,88,92,95]. Neutrophils then move toward the site of infection/inflammation through chemotaxis, guided by a chemotactic gradient of signals such as chemokines (e.g., IL-8), bacterial products (e.g., fMetLeuPhe), and complement factors. Neutrophil chemotaxis is characterized by three different processes: gradient sensing, polarization, and cell motility. Neutrophils sense these signals via surface receptors, triggering intracellular pathways that reorganize their cytoskeleton. PI3K and MAP kinase-dependent pathways have a critical role in neutrophil chemotaxis. These signaling pathways contribute to asymmetric F-actin polymerization, where the leading edge extends toward the gradient of chemoattractants using actin polymerization, while the rear contracts to propel the cell forward. This directed amoeboid movement ensures neutrophils efficiently reach their target [96,97].

Organ-specific endothelial cell heterogeneity (Figure 1) results in organ-specific expression of adhesion molecules and receptors, and this heterogeneity plays an important role in tissue homeostasis and regulation of neutrophil–endothelial cell interaction [45,49,73,81,98,99]. For example, lung endothelial cells typically express higher levels of adhesion molecules, which facilities neutrophil adhesion and migration. Neutrophil recruitment in liver sinusoids’ endothelium is independent of selectins and rolling [81,100]. While in glomerular capillaries of the kidney, neutrophils are captured with the assistance of monocytes and platelets. Instead of rolling, they adhere to the endothelial cells through involvement of selectins, ICAM-1, PSGL-1, and integrins [81,101]. In contrast, brain endothelial cells in the blood–brain barrier (BBB) restrict neutrophil extravasation due to unique properties, including tight junctions supported by pericytes and astrocytes and to low expression of adhesion molecules [45,49,98]. This organ-specific heterogeneity leads to diversity in neutrophil–endothelial cell interaction and in the response to sepsis and severity of organ injury with the lung, kidney, and liver, the most sensitive organs [102].

**Figure 3:**
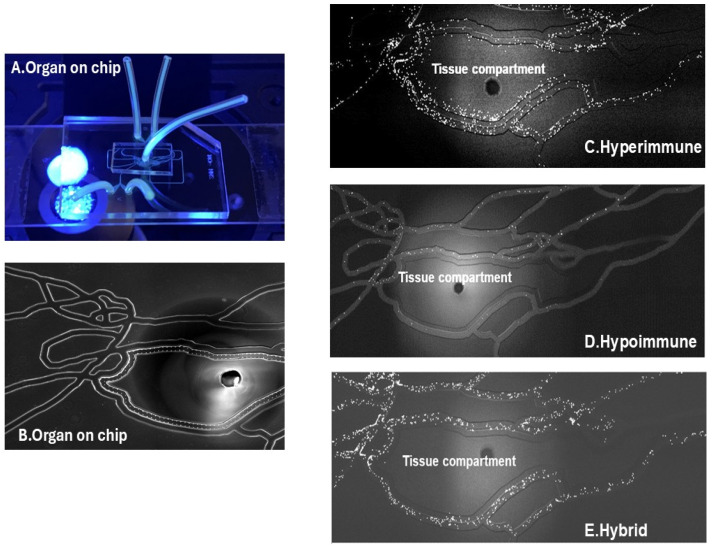
Organ-on-chip design (**A**) The image shows the vascular channel network and inlets and outlets with tubing inserted. (**B**) Bright field image shows the vascular channels lined by human lung microvascular endothelial cells and tissue compartment of the organ-on-chip. Neutrophil adhesion and migration patterns identify distinct neutrophil functional phenotypes labeled as Hyperimmune, Hypoimmune and Hybrid. (**C**) Hyperimmune: representative functional response of Hyperimmune neutrophils to cytomix in the organ-on-chip, demonstrating increased neutrophil adhesion in the vascular channels and increased migration across human endothelial cells into the tissue compartment. (**D**) Hypoimmune: representative functional response of Hypoimmune neutrophils to cytomix in the organ-on-chip, demonstrating decreased neutrophil adhesion in the vascular channels and decreased migration across human endothelial cells into the tissue compartment. (**E**) Hybrid: representative functional response of Hybrid neutrophils to cytomix in the organ-on-chip, demonstrating increased neutrophil adhesion in the vascular channels and decreased migration across human endothelial cells into the tissue compartment. Reproduced with permission from authors (published in *Frontiers in Immunology*, 7 March 2024, Volume 15, https://doi.org/10.3389/fimmu.2024.1341752) [38].

Neutrophil heterogeneity in sepsis also plays an important role in neutrophil–endothelial cell interaction and neutrophil migration. While increased adherence and migration are well described [38,103], immunocompromised neutrophils with impaired function have also been reported in sepsis patients [4,16,22,23,24], as well as phenotypes with mixed responses [26,27]. Impaired neutrophil migration has been described in sepsis where neutrophils accumulate inside the lumen of vessels, and decreased migration to the target tissue can occur following an increase in adhesion to endothelial cells, as their deformability is altered. Proinflammatory mediators, like fMetLeuPhe or TNF, stimulate the accumulation of F-actin in the cell membrane via a mechanism dependent on peroxisome proliferator-activated receptor gamma (PPARγ), resulting in decreased cellular deformability [104,105]. While the efficiency of neutrophils’ rolling is reduced due to shedding of selectins in sepsis, dysregulation of CD11a/CD18, downregulation of CXCR2, and upregulation of CXCR4 further disrupt neutrophil migration [17,25]. Insufficient migratory response of neutrophils contributes to their impaired infection control in severe sepsis and to ischemic damage caused by vessel occlusion by sequestered neutrophils on the endothelial barrier [25,106]. In a recent study of ICU patients with severe sepsis, we identified three distinct functional phenotypes of neutrophils based on their ex vivo neutrophil–endothelial cell interactions and neutrophil migration patterns [38] (Figure 3). Using a novel microphysiological system, as described in Section 6, we defined these three functional phenotypes as (1) Hyperimmune (characterized by enhanced neutrophil adhesion and migration), (2) Hypoimmune (unresponsive to inflammatory stimulation with decreased adherence and migration), and (3) a Hybrid or mixed phenotype that demonstrated increased adhesion but impaired migration. Thus, the heterogeneity of neutrophil phenotypes in sepsis significantly impacts neutrophil–endothelial cell interactions, resulting in very different patterns of adhesion, migration, and vascular barrier permeability.

During sepsis, in addition to regulating trafficking to vital organs, neutrophil–endothelial cell interaction can also lead to significant endothelial cell damage indirectly through the release of mediators and by direct contact. Excessive NET formation is a hallmark of dysregulated immune response that can impair endothelial barrier integrity [107,108]. NET histones, which are modified post-translationally by peptidyl arginine deiminase 4 (PAD4), are cytotoxic and can lead to endothelial cell death by membrane disruption. Enzymes in NETs impair endothelial cell function and integrity. Neutrophil elastase regulates adhesion molecules, such as VE cadherin (vascular endothelial cadherin) expression in endothelial cells, leading to disruption of cell junctions [108,109,110,111]. Components of NETs, including MPO, can lead to glycocalyx shedding, resulting in the exposure of more adhesion molecules on endothelial cells and increased neutrophil recruitment from the blood stream, neutrophil trafficking, and vascular permeability, producing tissue and organ damage [46,74,112,113].

Thus, with the identification of multiple distinct neutrophil phenotypes, it is becoming increasingly apparent that in sepsis, neutrophils are not a uniform cell population with vastly different mechanisms of interactions with endothelial cells. With this diversity, future drug development should be tailored to focus on different phenotypes and their specific cell functions, such as NETs, rather than global immunosuppression. Furthermore, the diversity of organ-specific endothelial cells suggests that unique therapeutic targets may be required for different organs.

## 5. Neutrophil Reverse Migration: Contributing to Heterogeneity

Reverse-migrated neutrophils refer to the phenomenon when neutrophils, after migrating to and performing their functions at the site of inflammation or infection, migrate back into the circulation instead of undergoing local apoptosis and clearance by macrophages [34]. This process has gained attention in studies of the pathophysiology of sepsis due to its possible implications for prolonging and disseminating the inflammatory response and progression of the disease to distant organ damage as, for example, recruitment of reverse-migrated neutrophils has been reported to cause lung damage [36,114]. The ultimate purpose and impact of reverse-migrated neutrophils is not well understood. Whether they cause inflammation progression or, by traveling away from the inflammatory foci, modulate the resolution of inflammation is still unknown.

While several potential underlying mechanisms of neutrophil reverse migration have been suggested, the precise mechanisms governing reverse migration have yet to be fully elucidated. Current evidence suggests a complicated interplay of neutrophil–endothelial cell interaction, adhesion molecules, intracellular signaling pathways, and chemokines are required for reverse migration to occur. Reverse-migrated neutrophils display a distinct phenotype characterized by altered surface markers and functional properties [115]. These neutrophils often express elevated levels of ICAM-1, show increased production of ROS and NETs, and demonstrate greater rigidity and prolonged survival due to reduced apoptosis (Figure 2). In addition, these neutrophils have reduced expression of chemokine receptors such as CXCR1 and CXCR2, as well as decreased L-selectin (CD62L) and increased β2 integrin (CD18). These changes collectively contribute to their tendency to adhere to the endothelial surface and their limited ability to re-enter tissues. Despite the well-known role of endothelial cell ICAM-1 in facilitating neutrophil–endothelial interactions through CD11a/CD18 and CD11b/CD18, the function of increased ICAM-1 expressed on reverse-migrated neutrophils remains uncertain, since its ligands are absent on endothelial cells [116,117]. Additionally, neutrophils with high ICAM-1 expression are linked to delayed apoptosis and are often elevated in systemic inflammatory diseases such as atherosclerosis and rheumatoid arthritis [118]. In neutrophils recruited in response to TNF and LPS, the integrins CD11a/CD18 and CD11b/CD18 prevent initiation of the caspase cascade, resulting in delayed apoptosis of transendothelial migrated neutrophils, most likely via ERK and PI3K-Akt activation [119,120]. After reverse migration, neutrophils become adherent to endothelial surfaces in distant organs, rarely rolling or migrating further, which can lead to endothelial barrier damage and tissue injury due to their intravascular accumulation.

Chemokines and other directional signals play a key role in neutrophil movement, and the process of reverse migration is influenced significantly by changes in chemokine gradients. Normally, neutrophils are drawn toward inflamed tissue by a chemoattractant gradient; however, when inflammation resolves and these gradients diminish, neutrophils may reverse-migrate. Additionally, receptor desensitization caused by excessive or prolonged stimulation during chronic inflammation can also lead to misdirected migration [121,122]. Specific chemoattractants such as leukotriene B (LTB4) not only promote neutrophil activation but also enhance elastase production, which can cleave JAM-C, a junctional adhesion molecule, thereby compromising endothelial cell integrity and promoting reverse migration [123]. Lipid mediators involved in inflammation resolution, such as lipoxin A4 (LXA4), promote neutrophil reverse migration in vitro [83,124]. Other molecules such as chemerin, acting through atypical receptors CCRL2, have also been implicated in facilitating neutrophil reverse migration from inflammatory sites [125]. Moreover, the protease cathepsin C, through activation of neutrophil serine proteases such as elastase, may support reverse migration by enabling extracellular matrix remodeling [34]. Interestingly, hypoxia-inducible factor (HIF) can inhibit neutrophil reverse migration while promoting apoptosis [126].

Additionally, mechanical factors such as shear stress play an important role in the behavior of reverse-migrated neutrophils. Shear forces increase integrin affinity, clarifying why neutrophils often crawl against the direction of blood flow along the endothelium during both forward and reverse migration [95]. Reduced expression of CXCR1/2 and increased rigidity contribute to the inability of reverse-migrated neutrophils to transmigrate across inflamed endothelium or navigate microvasculature, causing them to accumulate intravascularly, which, in turn, damages endothelial barriers in distant tissues. Disruption or downregulation of JAM-C on endothelial cells further facilitates reverse-migrated neutrophils through paracellular routes. For example, knockdown of JAM-C in endothelial cells can promote neutrophil movement in the reverse direction [114,127].

Mitochondrial function is disrupted in sepsis [128]. Itaconate is synthesized in the Krebs cycle by the enzyme aconitate decarboxylase (ACOD1 product of immune response gene 1 (IRG1)), which has been reported to have immunomodulatory effects in different inflammatory settings and anti-inflammatory effects in sepsis [129,130]. Neutrophils express itaconate in response to bacterial infection, and itaconate reduces bactericidal activity and impairs survival [131,132]. Itaconate release by macrophages and its related gene expression and derivates impact neutrophil migration. IRG1, which encodes the enzyme producing itaconate, plays a role in regulating neutrophil recruitment during infection. Itaconate is known to suppress NETs’ release, yet reverse-migrated neutrophils paradoxically show increased expression of IRG1, the gene encoding the enzyme responsible for itaconate production [133]. This raises unresolved questions: Is the effect of itaconate on reverse-migrated neutrophils independent of its role in inhibiting NETs’ formation? Or do neutrophils migrate in reverse because they are less prone to forming NETs? The relationship between itaconate and reverse-migrated neutrophils requires further confirmation. Increased IRG1 expression and concurrent decrease in neutrophil ICAM-1 at sites of inflammation are observed in association with itaconate [133]. In contrast, during ischemia–reperfusion injury, reverse-migrated neutrophils can induce inflammation in remote organs, marked by high ICAM-1 expression and enhanced ROS production, both contributing to intensified interactions with endothelial cells and secondary organ damage.

Thus, reverse migration is a multifactorial process involving dynamic interactions between neutrophils and the endothelium, guided by alterations in surface markers, chemokine gradients, biophysical forces, and other regulators such as itaconate. Many mechanistic aspects remain unclear, highlighting the need for further research into the regulation of neutrophil phenotypes, endothelial crosstalk, and molecular pathways involved in reverse-migrated neutrophils (Table 1).

## 6. Emerging Methodologies to Evaluate Neutrophil–Endothelial Interaction

Recently, the NIH announced a major new initiative to prioritize human-based research and decrease funding for animal-only studies. This new initiative places a greater emphasis on using NAMs (new approach methodologies), which include microphysiological systems (organ-on-a-chip) and organoids [134]. Microphysiological systems (MPSs) technology enhances the ability to model human biology in ways that animal models cannot achieve. Recently, the FDA Modernization Act 2.0 (2022) and Modernization Act 3.0 (2025) enable the FDA to use advanced non-animal methods such as MPSs to assess drug safety and efficacy. Further, a recent article in *Science* [135] highlights the shift in research away from animal models to NAMs.

Researchers have employed a variety of novel methods to study neutrophil–endothelial cell interactions. Cell culture models are often used to investigate responses to infection and inflammation but have limitations in the study of cell–cell interactions and leukocyte migration, as they do not generally involve 3D tissue architecture containing multiple cell types or modeling the shear forces in the vascular system [39]. Rodent and zebrafish models have been used to study neutrophil migration. However, the validity of animal models for studies of neutrophil–endothelial cell crosstalk during sepsis has been questioned due to the low levels of circulating neutrophils in rodents and significant differences between inflammatory mediators in rodent models of sepsis compared to humans [136]. Although zebrafish models have significantly advanced our understanding of neutrophil function, their relevance for studying human disease is restricted, as the similarities between zebrafish and human neutrophil function are not well characterized, and few gene markers are available in zebrafish to define different functional states [137,138]. For example, studies using LPS in zebrafish reported changes in microvascular circulation and inflammatory mediators; however, these models often have non-specific inflammatory responses and high mortality rates due to difficulty in controlling the LPS dosage [139]. Moreover, the failure of clinical trials using therapies developed in sepsis animal models has brought into question the translatability of these observations from animals to humans [140]. Emerging cell functional analysis, omic and in silico modeling approaches, have focused on using human endothelial cells and neutrophils in MPSs coupled with proteomics, ribonucleic acid (RNA) sequencing, and flow cytometry. MPSs are useful for studying the immune system by mimicking structures like blood vessels and tissues. These devices provide a controlled environment and allow real-time observation of human neutrophils and endothelial cell interactions, neutrophil migration (both forward and reverse), NETosis, and interactions with other cells. For example, studies employing a microfluidic model were able to demonstrate factors involved in reverse-migrated neutrophils and the inhibitory effect of neutrophil phagocytosis on reverse migration [45,141,142,143].

We have employed a novel MPS to evaluate neutrophil rolling, adhesion, and migration in realistic microvascular networks surrounding a tissue compartment under physiological conditions, thus facilitating the assessment of all steps of the neutrophil–endothelial cell interaction during inflammatory conditions such as sepsis [39]. Using this assay, we quantified adhesion molecule expression, as well as endothelial barrier damage and permeability, to develop a better understanding of neutrophil–endothelial cell interactions in sepsis. Furthermore, MPSs can be used to study neutrophil heterogeneity by studying cell surface markers and functional evaluation of these cells (including phagocytosis, NETs’ formation, apoptosis, forward and reverse migration, and ROS generation). As described in Section 4, using a novel MPS, we identified three distinct functional neutrophil phenotypes (Hyperimmune, Hypoimmune and Hybrid) ex vivo in neutrophils obtained from patients with severe sepsis [38] (Figure 3).

**Figure 4:**
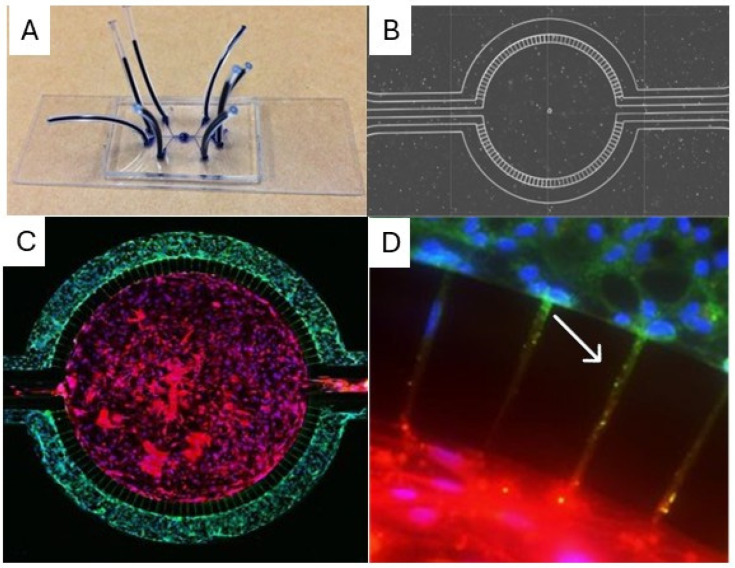
Blood–brain barrier organ-on-chip (**A**). Brightfield microscopic image demonstrating two vascular channels surrounding a tissue compartment (**B**). Cultured human brain microvascular endothelial cells in the vascular channel and astrocytes and pericytes in the tissue compartment (**C**). Endfoot of astrocyte (white arrow) communicating with brain endothelial cells (**D**). Adapted from Deosarkar, S. P.; Prabhakarpandian, B.; Wang, B.; Sheffield, J. B.; Krynska, B.; Kiani, M. F. “A Novel Dynamic Neonatal Blood-Brain Barrier on a Chip”. *PLoS One* 2015, 10 (11). https://doi.org/10.1371/journal.pone.0142725, with permission [145].

We have also employed MPS assays to test novel therapeutics for targeting neutrophil–endothelial cell interactions in sepsis. We found a protein kinase C delta (PKCδ) inhibitor, decreased ICAM-1 expression, and other adhesion molecules on endothelial cells in response to inflammation, leading to a reduction in neutrophil adhesion and migration across the endothelial cell barrier [144]. Similarly, the impact of organ-specific endothelial cell heterogenicity on their interaction with neutrophils can be studied using these MPSs. For example, blood–brain barrier MPS assays containing primary human brain endothelial and parenchymal cells mimic not only the three-dimensional morphology and flow characteristics but also the low barrier permeability characteristic of the blood–brain barrier in vivo [145] (Figure 4). We have used this blood–brain barrier MPS to show a protective effect of PKCδ inhibition from sepsis-induced vascular damage [146]. Lung endothelial cell dysfunction associated with sepsis-induced ARDS was modeled using an MPS assay comprised of lung endothelial cells treated with sepsis patient plasma [147]. Using this model, the authors found that sepsis patient plasma had a significant impact on lung endothelial cells, resulting in barrier dysfunction, increased adhesion molecule expression, and increased proinflammatory cytokine production.

Thus, MPSs offer a novel experimental platform to study the mechanisms of neutrophil–endothelial cell communication and the mechanisms underlying sepsis-induced endothelial cell dysfunction and to develop and screen therapeutics using primary human cells. Despite the enormous promise of MPSs, there are a number of challenges before MPS technology can reach its full potential. There is a need for manufacturing standardization, materials which don’t absorb small, hydrophobic drug molecules, and development of standard media containing nutrients and growth factors to support multiple cell types [148,149]. While MPSs provide important mechanistic information, these MPSs are not truly organ-on-a-chip, as they usually only contain a few organ-specific cell types and not all cell types needed to reproduce the structural and functional attributes of a single organ [148]. Another challenge of MPS models is the limited time of viability of cells in these models; this hinders the possibility of studying neutrophil–endothelial interaction over longer time periods to assess long term consequences [150,151,152]. To create a detailed sepsis model, multi-organ MPSs are needed to examine interactions with different organs, organ–organ communication, and the role of immune cells and other blood products.

Organoids are complex 3D cell culture systems that can be used to model organs [153,154]. Organoids are composed of stem cells derived from either human-induced pluripotent stem cells (iPSCs) or adult stem cells, organ-specific progenitor cells, an extracellular matrix (often Matrigel), and appropriate growth factors which support organoid growth and maturation [155,156]. Organoids and stem cell-derived models have been used to study sepsis-related organ damage in liver and intestinal epithelium, as they can mimic the structure and function of human organs in vitro [157,158]. Human lung organoids can serve as a realistic model to study cellular response mechanisms in infection [159]. Human embryonic stem cells, derived from early embryos (HESCSs), have been used to generate a lung organoid to study interaction of streptococcus pneumoniae with alveolar epithelium, including the host immune response [160]. Co-culturing of immune cells in organoids is a promising experimental approach to study immune cells’ interactions in infectious disease. Researchers have utilized organoids to study neutrophils’ interaction with intestinal epithelial in the setting of infection with salmonella [161]. Novel vascularized organoids have functional vasculature inside organoids to provide insights into immune cell trafficking and associated cytokines and their interaction within the heterogenous endothelial layer of different organs in sepsis [162,163,164]. However, organoids have shortcomings in establishing a complete model to fully mimic physiology, the immune microenvironment, and the complex microvascular network in the human body. This highlights the need for future research to improve their applicability in studying cell–cell interaction in sepsis and drug discovery.

Omic analysis, the comprehensive study of DNA, RNA, proteins, or metabolites, provides powerful tools for understanding the mechanisms of complex diseases. For sepsis, omic analysis can facilitate identification of prognostic and diagnostic biomarkers in clinical trials [165]. Integration of large volumes of data collected from clinical trials, experimental MPS, animal models, omic analysis, and cell culture into in silico models offers the intriguing possibility of developing a more complete comprehension of the underlying mechanism of sepsis-induced neutrophil–endothelial cell interaction and identifying novel treatment targets [39]. By combining these emerging methodologies, researchers gain novel insight on molecular approaches, intracellular signaling, gene translation, neutrophil phenotypes, functional alterations, and neutrophil–endothelial interactions. However, translation of omics findings to clinical use may be challenging, and there is a need to reach a standardized and rapid method to be practical in the clinical setting [62]. Future research using these combined methodologies may provide important insight into mechanisms of neutrophil–endothelial cell interactions and the impact of neutrophil and endothelial cell heterogeneity.

## 7. Therapeutic Innovation: New Targets for Treatment of Sepsis

Aberrant neutrophil–endothelial cell interactions have a critical role in the development of organ damage. Drugs reducing neutrophil adherence and migration, neutrophil release of toxic mediators including NETs, and protecting vascular endothelial cells are prime targets for preventing organ damage in sepsis. Therefore, targeting a specific neutrophil subtype or an explicit impaired activity in sepsis, such as interaction with endothelial cells or reverse-migrated neutrophils, may be a novel therapeutic approach.

### 7.1. Neutrophils as a Therapeutic Target

As neutrophil dysregulation is an important underlying component of sepsis, neutrophils may be a potential therapeutic target for modifying the immune response in sepsis with the goal of increasing their antimicrobial effects accompanied with better controlled release of NETs, reactive oxygen species, and proteases, as well as targeting forward and reverse migration to decrease their unwanted inflammatory dissemination and organ damage [117]. However, depletion of neutrophils while the primary infection is not yet under control worsens the outcomes, even though distal organ damage is reduced when neutrophils are depleted [25].

With the identification of neutrophil phenotypes [38,40], phenotype-specific drug targets need to be considered. In previous studies, we identified functional neutrophil phenotypes (Hyperimmune, Hypoimmune, and Hybrid) that expressed unique proteomic signatures indicating significant intrinsic differences in protein expression among these neutrophil functional groups [38]. To identify specific drug targets for these different neutrophil phenotypes, functional enrichment and protein–protein interaction network analyses were used to reveal key cellular pathways and examine differentially expressed protein (DEP) hubs that could serve as promising targets for therapeutic repurposing [40]. Importantly, several FDA-approved and experimental drugs were mapped to these targets, with specific examples including agents directed at Vitronectin (VTN) in the Hybrid phenotype, Transient receptor potential cation channel subfamily V member 2 (TRPV2) in the Hypoimmune phenotype, and H2A clustered histone 21 (H2AC21) in the Hyperimmune phenotype, highlighting potential avenues for phenotype-specific sepsis treatment [40]. This synergistic combination of MPS assays, clinical data, proteomics, and in silico modeling to provide important mechanistic insight into the interaction of different neutrophil phenotypes with endothelial cells in sepsis may provide a roadmap for differential treatment of sepsis neutrophil subpopulations and their response to therapeutics, leading to precision medicine (Table 2).

Targeting the reverse-migrated neutrophils as a potential determinant of organ damage might also control the dissemination of inflammation to other organs. However, targeting reverse-migrated neutrophils requires careful consideration of the appropriate timing in the course of the disease to avoid hindering the initial immune response to infection. In addition, induction of neutrophil apoptosis helps in the resolution of inflammation [166]. Studies focused on localized inflammatory diseases have targeted reverse migration to enhance inflammation resolution by, for example, inducing the reverse migration of neutrophils or neutrophil apoptosis (Table 2). Studies in zebrafish have shown that chlorogenic acid-enriched kudingcha extract promotes neutrophil reverse migration via phosphorylation of ERK and AKT [167]. In another study on wound repair using zebrafish larvae, the CXCR2 inhibitor SB225002 produced a dramatic retention of neutrophils at the wound site by blocking reverse migration [121]. However, the pathophysiology of sepsis differs from localized inflammation, and excessive neutrophil reverse migration could exacerbate systemic inflammation and distant organ damage in sepsis. Death receptor ligands and cyclin-dependent kinases (CDKs) play critical roles in regulating neutrophil apoptosis, which is a key process in the resolution of inflammation (Table 2) [168]. In sepsis, this tightly controlled mechanism is often disrupted, and reverse-migrated neutrophils exhibit delayed apoptosis, leading to prolonged survival and sustained inflammation [169]. Targeting death receptor ligands, such as the Cyclin-dependent kinase inhibitor AT7529, facilitated inflammation resolution by inducing neutrophil apoptosis in preclinical models of lung inflammation using the neutrophil of sepsis-related ARDS patients [170]. Another CDK inhibitor, R-roscovitine, in animal models of pleural and lung inflammation, and in arthritis, induced apoptosis in neutrophils to resolve chronic inflammation [171,172]. Both of these two CDK inhibitors induce cell cycle arrest and apoptosis; however, there are differences in their downstream mechanisms. AT7529 also inhibits transcription and activates glycogen synthase kinase- 3β, while R-roscovitine downregulates myeloid cell leukemia-1 [173,174]. CDK inhibitors merit preclinical and clinical study in sepsis to confirm the significance of the role of reverse-migrated neutrophils in sepsis pathogenesis.

### 7.2. Endothelial Cells as Therapeutic Targets

Endothelial cells also represent an important therapeutic target in sepsis (Table 2). For example, impaired VE-Cadherin expression in sepsis can be targeted with proprotein convertase subtilisin kexin 9 (PCSK9) inhibitors [175]. A systematic review and meta-analysis reported no association between PCSK9 inhibitors with risk of incident sepsis and severe infections [176]. Alirocumab, a PCSK9 inhibitor, is currently under evaluation in a phase Ib clinical trial for treatment of sepsis (NCT05469347) [177]. Glycocalyx protective therapeutics, such as inhibition of glycocalyx shedding by heparin [178], may also serve as a potential approach to maintain endothelial cell integrity. Statins are another potential therapeutic with multiple mechanisms of action that could target neutrophil–endothelial interaction through the inhibition of NFκB and dampening proinflammatory signaling [179]. Statins have been studied in different clinical trials for treatment of sepsis, septic shock, and acutely injured lungs from sepsis. A clinical trial studying septic shock (NCT02681653) reported atorvastatin induced-changes in inflammatory biomarkers, including IL-1, IL6, TNF-α, IFN, and CRP; however, no mortality benefit was reported [180]. Another clinical trial studied atorvastatin therapy in severe sepsis and reported no effect on IL-6 levels. (ACTRN 12607000028404) [181]. Rosuvastatin did not improve clinical outcomes in patients with sepsis-associated ARDS. (NCT00979121) [182].

### 7.3. NETs and Other Neutrophil Bactericidal Mediators as Therapeutic Targets

Reducing the destructive effects of neutrophil products on endothelial cells should also be considered as a potential therapeutic target. For example, antioxidants, which inhibit the oxidative burst in neutrophils and therefore limit ROS production, or metalloproteinase inhibitors, which might limit the neutrophil migration, are among proposed therapeutic targets for sepsis [183]. As NETs damage vascular endothelial cells, therapies targeting NETs’ formation, like DNase in combination with antibiotic therapies (metronidazole and cefuroxime), have been reported to produce better outcomes in preclinical models [184,185]. Efficient cleavage of NETs requires combined action of two types of DNases, DNase 1 and DNase1 like 3, which degrade naked dsDNA and chromatin, respectively. A phase I clinical trial is currently underway evaluating IV DNase I in ICU septic patients (NCT05453695) [186]. In an animal study, reparixin, a CXCR1/2 inhibitor which could also have a potential therapeutic effect targeting reverse migration, reduced NETs formation and reduced multiorgan injury and mortality in septic mice without any impairment in bacterial clearance [187]. While only studied preclinically in sepsis, reparixin has been evaluated in phase II and III clinical trials for ARDS and COVID-19 pneumonia, respectively [188,189]. Reparixin showed a trend toward limiting disease progression as an add-on therapy in COVID-19 severe pneumonia (NCT04878055); the results of the ARDS clinical trial have not yet been reported (NCT05496868) [190]. Inhibition of peptidyl arginine deiminase 4 (PAD4), an enzyme that catalyzes post-translational histone modification in NETs’ formation, increased survival in murine sepsis models [191]. Simvastatin reduces NETs’ formation via PAD4 in vitro [192]. A reduction in neutrophil NETosis in sepsis patients with pneumonia following simvastatin therapy was associated with improved clinical outcomes, including improved Sequential Organ Failure Assessment (SOFA) scores and hospitalization-free survival [193]. However, the timing and scope of NETs’ inhibition to allow the neutrophils to perform their primary antimicrobial duty is not fully understood, as NETs have an important role in the antibacterial innate immune response [194]. In sepsis, early NET inhibition increases bacterial load, whereas it could be beneficial in later stages of the disease. It should be noted that most preclinical studies used stimulants such as PMA (phorbol 12-myristate 13-acetate) to induce NETs’ formation, which does not accurately reflect the numerous triggers of NETosis in vivo. Further studies are needed to address the gaps in knowledge of inhibition of NETs on clinical outcomes and to standardize this approach to reduce adverse effects.

**Table 2:** Therapeutic targets for sepsis treatments: focusing on dysregulated neutrophil–endothelial interaction.

Therapeutic Targets	Examples	Mechanism of Action	Potential Applications	Preclinical/ Clinical Status
**Neutrophils**	CDK inhibitor (R-roscovitine, AT7519)	Induction of neutrophil apoptosis	Resolution of chronic inflammation	Preclinical: animal and ex vivo patient neutrophil studies [172,173,174]
	VTN TRPV2 H2AC21	Targeting different neutrophil functional phenotypes	Precision medicine in the treatment of sepsis by targeting neutrophil phenotypes	Preclinical [40]
	SB225002	CXCR2 inhibitor	Inhibiting reverse neutrophil migration	Preclinical: (animal study) [123]
**Endothelial cells**	PCSK9 inhibitor (alirocumab)	Increased expression of the endothelial cellular junction adhesion molecule VE-Cadherin	Prevention of PCSK9-induced vascular endothelial cell injury in sepsis	Phase Ib clinical trial (NCT05469347) [179]
	Statins (atorvastatin, rosuvastatin)	NFκB inhibitor Multiple targets	Prevention of proinflammatory reprogramming of endothelial cells	Phase II clinical trials (NCT02681653) [182] (ACTRN 12607000028404) [183] (NCT00979121) [184]
**NETs**	DNases	Cleavage of NETs	Decrease NET-induced damage to vascular endothelial cells in sepsis	Phase I clinical trial (NCT05453695) [188]
	Reparixin	IL-8 receptors (CXCR1 and CXCR2) inhibitor	Reduction of NET production and decreased thrombosis and organ injury. Also may inhibit neutrophil reverse migration.	Preclinical for sepsis Clinical trial, phase III, for COVID-19 pneumonia (NCT04878055) [191,192] Clinical trial, phase II, for adult patients With ARDS (NCT05496868) [190]

## 8. Conclusions and Future Directions

Sepsis is characterized by life-threatening organ dysfunction caused by a dysregulated host response to infection. Neutrophils are key contributors to the dysregulated immune response and play a critical role in vascular barrier damage and development of organ failure. In order to develop new drugs, a better understanding of the fundamental mechanisms of neutrophil–endothelial interactions in sepsis is needed. In our view, it is critical to appreciate that distinct neutrophil phenotypes interact differently with endothelial cells and have diverse patterns of adherence and migration/reverse migration and differential patterns of organ-specific endothelial cell activation. Furthermore, the critical contribution of NETs to endothelial cell damage and organ failure is becoming well appreciated and has emerged as an important therapeutic target in sepsis. The importance of neutrophil reverse migration in sepsis is also emerging as a potential target in drug development. The context-specific function of reverse neutrophil migration needs to be considered not only because it plays a critical role in local inflammation but also because it often triggers systemic damage in secondary organs. Development of therapeutics specific to different neutrophil phenotypes in sepsis requires a synergistic understanding of neutrophil function heterogeneity in different phenotypes, of the role of neutrophil reverse migration, and of organ-specific endothelial cells. Developing this synergistic understanding has been limited by a lack of translatable physiologically relevant models. Emergence of NAMs, such as microphysiological systems and organoids employing primary human cells along with in silico models, provides a unique opportunity to develop and screen therapeutics that are more likely to be clinically relevant.

Future studies should focus on standardizing neutrophil phenotyping by using a multipronged approach which accounts for not only variations in cell markers and protein expression but also functional changes. It is important to ascertain whether neutrophil heterogeneity in sepsis is dependent on disease stage and evolves over time or whether it is an intrinsic property of the patient’s immune function and reflects the heterogeneity and diversity of host responses. Understanding how these different phenotypes impact neutrophil–endothelial cell interactions and neutrophil migration is critical for targeted drug development. Similarly, NAM-related platforms and methodologies should be standardized so studies from different research groups can be directly compared, providing critical information for mechanistic studies of cell–cell communication and for drug development leading to precision medicine.

## Figures and Tables

**Table 1:** Neutrophil Forward vs. Reverse Migration.

	Forward Migration	Reverse Migration
**Direction**	From circulation into tissue	From tissue back to the circulation
**Initiation**	Sensing signals at the infection/inflammation site (e.g., PAMPs and DAMPs, chemokines, cytokines and other inflammatory mediators)	Not fully delineated. Proposed triggers and mechanisms include: diminished chemoattract gradient, receptor desensitization, lipid mediators (LTB4), and breakdown of junctional adhesion molecules
**Purpose**	Defense against pathogen and initiation of the inflammatory response	Facilitate the resolution of inflammation by removing migrated neutrophils from the site of infection/ inflammation
**Potential outcome**	Pathogen clearance and tissue repair, but if not adequately regulated, can cause tissue damage and organ failure	Dual role: Resolution of inflammation or if not adequately regulated, can contribute to the spread of systemic inflammation
**Surface markers**	Highly variable based on the phenotype and maturation/activation status of neutrophils (e.g., low ICAM-1, high CXCR1)	High ICAM and ẞ2 integrin Low CXCR1, CXCR2 and L- selectin
**Other Cellular Characteristics**	Highly variable based on the phenotype and maturation/ activation stage of neutrophils	Delayed apoptosis Increased rigidity Increased production of ROS and NETS

## Data Availability

No new data were created or analyzed in this study.

## Abbreviations

The following abbreviations are used in this manuscript:

ACOD	Aconitate decarboxylase
APC	Activated protein c
CCRL	C-C motif chemokine receptor-like
CD	Cluster of differentiation
CDK	Cyclin dependent kinase
CSF3R	Colony stimulating factor 3 receptor
CXC	Chemokine (C-X-C motif)
CXCL	Chemokine (C-X-C motif) ligand
CXCR	CXC chemokine receptor
DAMPs	Damage-associated molecular patterns
DARPA	Defense Advanced Research Projects Agency
DEP	Differentially expressed protein
DNA	Deoxyribonucleic acid
EPA	Environmental Protection Agency
FDA	Food and Drug Administration
FMLP	*N*-Formylmethionyl-leucyl-phenylalanine
H2AC21	H2A clustered histone 21
HDN	High-density neutrophil
HIF	Hypoxia inducible factor
ICAM	Intercellular adhesion molecule
IRG1	Immune response gene
JAM	Junctional adhesion molecule
LDN	Low-density neutrophil
LFA	Lymphocyte function-associated antigen
LPS	Lipopolysaccharide
LTB	Leukotriene B
LXA	Lipoxin A
Mac	Macrophage-1 antigen
MAPK	Mitogen activated protein kinase
MMP9	Matrix metalloproteinase-9
MODS	Multiple organ dysfunction syndrome
MPSs	Microphysiological systems
NAMs	New approach methodologies
NASA	National Aeronautics and Space Administration
NCATS	National Center for Advances of Translational Sciences
NET	Neutrophil extracellular traps
NF-κB	Nuclear factor kappa-light-chain-enhancer of activated B cells
NIH	National Institutes of Health
OI	Octyl itaconate
PAD4	Peptidylarginin deiminase 4
PAMPs	Pathogen molecular patterns
(PCSK9)	Proprotein convertase subtilisin kexin 9
PECAM	Platelet endothelial cell adhesion molecule
PI3K	Phosphatidylinositol 3 kinase
PKC δ	Protein kinase C delta
PPARγ	Peroxisome proliferator-activated receptor gamma
PSGL	P-selectin glycoprotein ligand-
RNA	Ribonucleic acid
ROS	Reactive oxygen species
TNF	Tumor necrosis factor
TRPV2	Transient receptor potential cation channel subfamily V member 2
VCAM	Vascular cell adhesion molecule
VEcadheri	Vascular endothelial cadherin
VLA	Very late antigen-
VTN	Vitronectin
